# Developing a Flexible Pediatric Dosage Form for Antiretroviral Therapy: A Fast-Dissolving Tablet

**DOI:** 10.1016/j.xphs.2017.05.004

**Published:** 2017-08

**Authors:** Manjari Lal, Manshun Lai, Marcus Estrada, Changcheng Zhu

**Affiliations:** PATH, PO Box 900922, Seattle, Washington 98121

**Keywords:** drug delivery systems, freeze-drying/lyophilization, HIV/AIDS, micelle, pediatric, solid-dosage form, tablet, ARV, antiretroviral, CMC, carboxymethyl cellulose, FDT, fast-dissolving tablet, LPV/r, fixed-dose combination of lopinavir and ritonavir, ODT, orally disintegrating tablet, RH, relative humidity

## Abstract

Current presentations of the anti-HIV drugs lopinavir and ritonavir make appropriate dosing for children difficult. We conducted a feasibility study to develop a formulation for these drugs with child-safe excipients in a flexible dosage form for children across the pediatric age spectrum. The freeze-drying in blister approach was used to produce fast-dissolving tablets (FDTs), as these can be dispersed in fluids for easy administration, even to infants, and appropriate portions of the dispersion can be given for different ages/weights. We combined various ratios of polymers, surfactants, and bulking agents to incorporate the 2 highly hydrophobic drugs while maintaining drug stability, rapid disintegration, and good handling properties. The final FDT was robust and disintegrated in 0.5 mL of fluid in 10 s with up to 4 tablets dissolving in 2 mL to achieve varying doses accommodated in a common teaspoon. Drug recovery after dissolution in small volumes of liquid or fluid foods was 90%-105%. The final candidate FDT was stable at 40°C, 75% relative humidity for up to 3 months. FDTs are a promising flexible dosage form for antiretroviral treatment for pediatric patients, especially in low-resource settings.

## Introduction

In 2015, an estimated 1.8 million children younger than 15 years were living with HIV/AIDS worldwide, with most residing in sub-Saharan Africa.^1^ Because of the lack of suitable pediatric antiretroviral (ARV) formulations, caregivers in low-resource settings often divide adult pills or crush them for administration to children.^2^ However, crushing has been reported to reduce absorption and change the pharmacokinetics of solid-format ARV medications,^3^ and this, along with difficulty estimating the amount of crushed material to give to a child, is likely to cause over- or underdosing. An age-appropriate dosage form with satisfactory characteristics regarding safety, acceptance, and convenient administration is needed for children, especially for the lowest age group (<12 months of age).

Although solid-format medications are convenient, allowing accurate dosing and small packaging volume, newborns and infants are developmentally unable to swallow most tablets. One study that investigated children aged 6 months to 6 years found that even those in the youngest group (6-12 months) could swallow one 2-mm tablet with up to 3 mouthfuls of liquid.^4^ This is encouraging information; however, only 10 children were tested in the age group, and 3 of them chewed the tablet. As noted above, many solid-format ARV medicines may not be crushed. Another question is how many tablets of this size would be needed to administer an adequate dose of an ARV formulation. Syrup-based and suspension formulations are easier to administer, but bottles of liquid drugs can be difficult to transport, and families struggle with refrigerating and measuring liquids.^5^ The WHO recommends dosage forms that are appropriate for developing countries and further prioritizes flexible solid-dosage forms such as tablets that are orodispersible or that can be prepared as oral liquids for younger age groups.^6^

The fixed-dose combination of lopinavir and ritonavir (LPV/r) at a ratio of 4:1 (e.g., 80 mg/20 mg) is recommended by WHO as first-line ARV therapy for HIV infected infants and children between 14 days and 3 years of age.^7^ Tablets are available for children weighing 14-25 kg who are able to swallow them, and a liquid formulation is approved for use in infants as young as 14 days; however, it contains excipients that raise safety concerns for use in the targeted age group, namely ethanol and propylene glycol.^8^, ^9^

In 2015, LPV/r 40 mg/10-mg heat-stable oral pellets contained in a capsule received tentative approval from the US Food and Drug Administration^10^ for use in children at least 14 days of age and weighing more than 5 kg. These oral minipellets are reported to be appropriate for even small children, as they allow giving the drug with soft food^11^; however, difficulty in opening the capsule could cause spilling of the pellets, the pellets cannot be stirred or crushed in food,^12^ and they must be consumed immediately. These are major limitations, especially for children <6 months of age, who may have difficulty consuming the entire food and minipellet content immediately, without chewing or crushing, to achieve a complete dose.

Another potentially adaptable solid-dosage form for pediatric ARV medication is the fast-dissolving tablet (FDT), produced by freeze-drying (lyophilization), an established process in the pharmaceutical industry. The applications of freeze-drying include manufacturing of rapidly disintegrating dosage forms, such as orally disintegrating tablets (ODTs), wafers, and thin films.^13^ ODTs are highly porous, resulting in dispersion within 5 s, but they are extremely fragile and require specialized packaging. Our laboratory has developed formulations for freeze-drying FDTs for vaccines that produce robust tablets yet maintain the fast-dissolving characteristic.^14^, ^15^ An additional challenge in developing a suitable pediatric formulation as an FDT for LPV/r is that these drugs are highly hydrophobic, and excipients to solubilize them for the formulation must be safe for children.

This article reports a feasibility study for developing an FDT containing highly hydrophobic ARVs for HIV treatment in children, especially in low-resource settings. Our goal was to produce an FDT containing a fixed-dose combination of LPV/r using safe excipients, without the aid of organic solvents. The FDT would have excellent physicochemical properties and would disperse in a minimal amount of aqueous fluid, for easy administration to children and adolescents from 14 days to 18 years of age.

## Materials and Methods

### Formulation Preparation

Mannitol, carboxymethyl cellulose (CMC), and Tween 20® were from Sigma-Aldrich (St. Louis, MO), and nonfat dry milk was from local Safeway stores (Seattle, WA; manufacturer: Safeway, City of Commerce, CA). Lopinavir and ritonavir were from L.G.Pharma (Saint-Laurent, QC). Excipients were added slowly to deionized water (ELGA® PURELAB® Classic system; High Wycombe, Bucks, UK) while stirring, in the following order: 1.8 wt% nonfat dry milk, 9 wt% mannitol, and 0.5 wt% Tween 20. CMC at 0.2 wt% was added slowly in small portions while stirring to avoid clumping of solid particles, which are very difficult to dissolve. The last step was the addition of LPV/r solid powder while stirring at 1200 rpm for 5 h, until a homogeneous milky emulsion was obtained, containing no solid particles. The concentration of LPV/r was 80 mg/20 mg per mL for the 2 drugs, respectively, in the final formulation.

Blister sheet forming and sealing were performed on a blister packaging machine (B2FS; Applied Engineering Corporation, Newark, NJ). Polyvinyl chloride blister sheets (Delta Circle Industries, Richmond, VA) were thermoformed to obtain 1-mL cavities with a diameter of 15 mm with the following parameters: preheat time 3.5 s, forming time 5 s, sealing time 3.5 s, set temperature 150 ± 5°C, and air pressure 80 psi.

### Freeze-drying

Lyophilization was performed as reported in our previous work.^14^ One milliliter of formulation emulsion was loaded into each cavity in the preformed blister sheets using a HandyStep® Electronic Repeating Pipette (BrandTech® Scientific, Essex, CT), and sheets were placed on lyophilization trays and into a Millrock Laboratory Freeze Dryer (LD85; Millrock Technology, Kingston, NY) with the condenser temperature at 70°C and vacuum at 100 mTorr. The formulations were rapidly frozen to −45°C and held for 3 h, followed by primary drying from −40°C to +30°C over a period of 22 h. Secondary drying was at 30°C for 5 h.

FDTs in blister sheets were sealed using a B2FS thermoforming and sealing machine (Applied Engineering Corporation) at 155°C to seal on a peelable foil lid (Delta Circle Industries, Richmond, VA). The blister sheets were sealed in aluminum foil sachets (Pharmaceutical Packaging Services, Richmond, VA) with a Medivac sealer (Aline Heat Seal Corporation, Cerritos, CA). The final sealed FDTs were stored at 4°C until further testing.

### Drug Quantification

Quantification of the 2 drugs in FDTs was conducted by gradient reverse-phase HPLC (Thermo Fisher Scientific^TM^ Dionex^TM^ UltiMate^TM^ 3000 series, Sunnyvale, CA), using an MWD-3000 UV-Vis detector (Thermo Scientific^TM^ Dionex^TM^) and a method based on the US Pharmacopeial Convention^16^ Pending Monograph for lopinavir and ritonavir tablets. The HPLC method was specific, and none of the formulation excipients interfered with the 2 analytes of interest.

Recovery of the 2 drugs was measured after dissolving FDTs in the following fluids (purchased in Seattle, WA): whole milk (Sunshine brand), 2% fat milk (Sunshine), applesauce (Gerber® 1st Foods®), and banana sauce (Kroger® Comforts for Baby® Stage 2 food).

### Drug Extraction

For extraction of LPV/r from food contents, acetonitrile and methanol (70:30) were added to the food in 20× dilution. The mixture was vortexed (Vortex-Genie® 2; VWR International, Radnor, PA) for 30 s, followed by centrifugation at 3310 rpm for 20 min (GPR Centrifuge; Beckman Coulter, Indianapolis, IN) to separate the food contents. Supernatants were collected and injected into the chromatographic system.

### Evaluation of Tablet Properties

Friability of the FDTs was tested using a drum modified from a sealed petri dish (VWR International) with a diameter of 6 cm, set to rotate at 50 rpm for 4 min, with rigorous conditions that are recommended for a harder tablet.^17^

The moisture content of the FDTs was determined by methanol extraction and coulometric Karl Fischer titration in a dry environment. Anhydrous methanol was added to each FDT, and the vessel was agitated for 1 min. The dry methanol was in contact with the FDT for 20 min and then was analyzed for moisture content using the C20 Karl Fischer Coulometric Titrator (Mettler-Toledo LLC, Columbus, OH).

Because the FDT dosage form is intended for administration as a fluid, a modified form of the US Pharmacopeial Convention disintegration test ‹701› method was used.^18^ A disposable syringe was used to deliver 0.5 mL of water directly onto a tablet placed on a flat surface. Completeness of disintegration of the tablet was checked by manual palpation of the tablet at the end of 30 s, which is set by the US Food and Drug Administration as the disintegration specification for ODTs.^18^

Dosing flexibility of FDTs was evaluated in a fixed volume of water. To simulate a typical in-home scenario, we used a tableware teaspoon that held approximately 4 mL of water. One FDT was dispersed in approximately 0.5 mL of water, and the drug content of LPV/r in the liquid was measured by HPLC. The measurement was repeated after dissolving up to 4 tablets in 2 mL of water.

For a stability study, sealed blister sheets containing LPV/r FDTs were stored in 40°C, 75% relative humidity (RH) chambers for 3 months. At each monthly time point, blister sheets were removed and tablets tested for recovery of LPV/r by HPLC.

## Results

**Figure 1:**
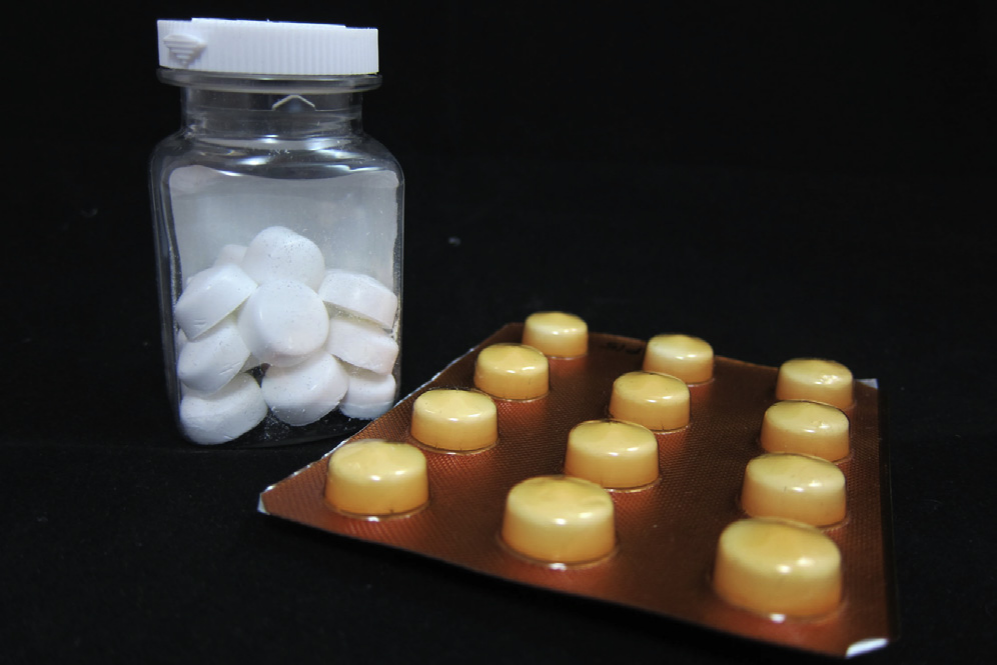
Lopinavir/ritonavir FDTs (Photo: PATH/Manshun Lai).

We screened 10 formulations combining various ratios of polymers, surfactants, and bulking agents for incorporating LPV/r at 80 and 20 mg/mL, respectively, to eventually produce a robust tablet while maintaining drug stability, rapid disintegration, and good handling properties (Supplementary Data). The final formulation composition and physicochemical properties of the FDT are shown in Table 1. These tablets were produced by freeze-drying the final liquid formulation in preformed blister sheets (Fig. 1). The tablets are robust and can be packaged and shipped in bulk in bottles, as well as in blister sheets.

**Table 1:** Composition and Properties of Lopinavir/Ritonavir FDT

Formulation Composition (wt%)	Appearance	Residue Upon Removal	Average Weight (mg)	Moisture Content (Weight % Water per Tablet)	Friability (% Weight Loss)	Average Disintegration Time (s)
Tween 80: 2.2% Nonfat dry milk: 8.8% Mannitol: 44.0% Carboxymethyl cellulose: 1.1% Lopinavir: 35.2% Ritonavir: 8.8%	Smooth, uniform, no chipping, or cracks	None	216	1.60	4.30	<10

**Figure 2:**
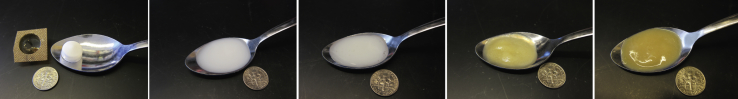
Disintegration of lopinavir/ritonavir FDTs in 2 mL of different fluids. From left to right: FDT; tablet dissolved in water, milk, applesauce, and banana sauce (All photos: PATH/Manshun Lai).

Post freeze-drying, the tablets were sturdy and easy to remove from blisters, with no or minimum residue and with good handling characteristics. The FDTs containing LPV/r disintegrated completely in <10 s in 2 mL of fluid or food, as shown in Figure 2.

The HPLC assay demonstrated that both lopinavir and ritonavir were recovered in the range of 90%-105% after disintegration in different fluids (data not shown).

**Figure 3:**
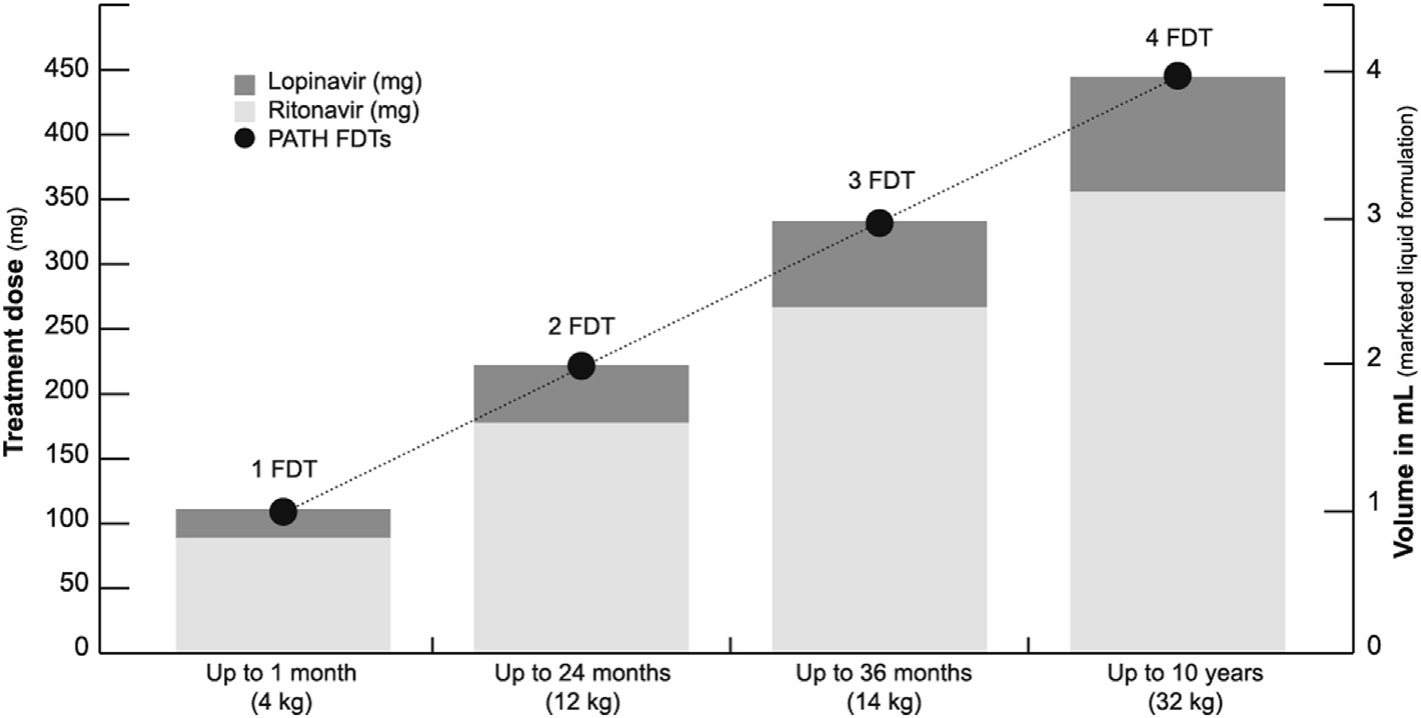
Dosing flexibility of lopinavir/ritonavir FDTs. Up to 4 tablets can be dissolved in 2 mL of water.

Adapted from: Jaclyn Delarosa/PATH.

Dosing flexibility was demonstrated by increasing the number of FDTs in a fixed volume (2 mL) of fluid, where the dose was verified after each FDT addition by measuring recovery of intact drugs via HPLC analysis for LPV/r. As shown in Figure 3, up to 4 FDTs can be dispersed in a volume of 2 mL, providing a dose of 320 mg/80 mg of LPV/r, adequate for a child up to 10 years of age.

Accelerated stability testing showed that FDTs in blister packaging did not undergo shrinkage or change in weight after up to 3 months of storage at 40°C, 75% RH. The tablets maintained good handling, with friability the same as before storage at approximately 4%. The disintegration time in water remained at <10 s at the end of the 3-month storage period, while the moisture content decreased from 1.6 wt% to 0.8 wt%. Recovery of intact LPV/r was within the acceptance criteria of 90%-105%, and HPLC profiles did not show any additional peaks, suggesting the absence of degradation products (data not shown).

## Discussion

As noted above, currently available ODTs developed using freeze-drying disperse rapidly in liquids but are fragile. The robust freeze-dried FDT we developed with an LPV/r has attributes of a solid-dosage form, including compact packaging and easy handling by caregivers. It also provides the advantages of a liquid formulation, because the highly porous structure allows rapid disintegration in a small volume of fluid for administration—which also permits dispensing portions of the fluid to provide appropriate doses for children of different weights. The use of freeze-drying for developing FDTs has some limitations. High drug loading in small volumes can result in a viscous formulation, which allows air bubbles to form during mixing and interferes with uniform filling of the blister cavities and nonuniformity in the final product. In addition, high levels of surfactants can cause foaming during lyophilization, resulting in spillage of material from blisters and loss of product. However, we did not encounter these problems in developing our FDT.

The 10 formulations tested in this study—using sugar, polymers, and surfactants as excipients—were based on PATH's prior experience in developing FDTs for vaccines.^14^, ^15^ Because the targeted levels of the combined drugs per tablet dose was high (∼100 mg), the 2 major criteria for down selection were drug-loading levels, based on analytical recovery, and physical properties of the tablet. Characteristics to be avoided in the physical properties of tablets were surface cracking or chipping, nonuniform appearance, extremely porous structure subject to static forces, residue left in blister cavities, and long dispersion times in fluid. The dosage form was being developed for pediatric use, so efforts were made to minimize the number of excipients and their levels in the final product, and those used are of “generally recognized as safe” quality and have a demonstrated acceptable safety profile for pediatric use.^19^ No preservatives were used in the formulations.

The purpose of the nonionic emulsifier Tween 20 was to minimize residue and assist in solubilizing the drug components. Mannitol was used not only as a crystalline bulking agent for imparting good handling properties, but also to add sweetness. CMC was a filler agent to add bulk and improve the overall handling properties of the FDT.

The composition of the formulation played a key role in solubilizing the hydrophobic drugs at high loading levels while maintaining rapid disintegration and good handling characteristics. Lopinavir and ritonavir are highly hydrophobic, with aqueous solubility of about 1 μg/mL and an octanol-water partition coefficient (log *P*) >4.^20^, ^21^ Nearly 50% of the weight of our tablet (∼210 mg) comes from the 2 drug components, which were solubilized at 80 and 20 mg/mL for lopinavir and ritonavir, respectively, in the presence of milk and polymer stabilizers. One explanation for the increased solubility (10,000-fold increase) in aqueous formulation could be the presence of surfactant combined with casein phosphoproteins in milk. Casein, an amphiphilic protein, may increase drug solubility through micellization, assisted by the Tween 20.^22^ Although we did not investigate the mechanism in our work, similar results for casein have been reported previously.^23^, ^24^, ^25^ Powdered milk has been used in approved veterinary vaccines,^26^ as part of a formulation for pediatric tablets produced by direct compression,^27^ and in production of freeze-dried solid dosage forms of viable *Lactobacillus* spp.^28^

After 3 months of storage at 40°C, 75% RH, tablet physical properties remained nearly the same as prior to storage, with friability and disintegration time unchanged. The decrease in moisture content from 1.6 wt% to 0.8 wt% may be a result of secondary drying at the elevated temperature, although this did not impact the handling properties of the FDT.

Each tablet disintegrated in just 0.5 mL of water, maximizing the likelihood of uptake of the full dose by small children, and there were no solid pieces that an infant might detect and expel. As many as 4 tablets (dose units) could be dispersed in volumes as low as 2 mL (<1 teaspoon), allowing a wide dosing range by simply increasing or decreasing the number of tablets in a fixed low volume. Furthermore, the low volume allows delivering the medication with a spoon, a commonly available implement in most households. Although we tested commercially produced foods, the tablets can be dispersed in milk or any available pureed food. No undesirable physical or chemical interactions between the food and the medicine were detected based on complete drug recovery using HPLC analysis. A question remaining is the taste of the product when dispersed in fluid and presented to children, as it is unclear whether the low volumes of fluid or food are sufficient to mask the bitter taste of the drugs. Palatability studies of the LPV/r FDTs in different fluids and infant foods in rodents have been completed and will be published separately. Rodents are useful models for taste studies^29^ based on analysis of lick patterns, and this study will determine whether the taste of the 2 drugs in the FDT dispersed in various foods is sufficiently aversive to alter the normal licking pattern.

These results indicate that the FDT offers flexibility for administering LPV/r across a broad range of age groups, and they suggest potential applicability for other combination ARV medications and for drugs for other disease indications in the pediatric population, especially in low-resource countries.
