# Supplementary material for: Developing a Flexible Pediatric Dosage Form for Antiretroviral Therapy: A Fast-Dissolving Tablet
Source: J Pharm Sci. 2017 Aug;106(8):2173–7. doi: 10.1016/j.xphs.2017.05.004 (PMC5514784; doi:10.1016/j.xphs.2017.05.004)
Supplement: Supplementary Table [file mmc1.docx]

**Table A. Composition of ten formulations tested for a fast-dissolving tablet for oral pediatric antiviral medications.*^a^***

| **Formulation**  **N = 2 replicates** | **Tween 20**  **wt%** | **MCC**  **wt%** | **NFDM**  **wt%** | **Mannitol**  **wt%** | **β-CD**  **wt%** | **CMC wt%** | **Tablet appearance** | **Blisters** | **DT (s)** | **Post-disintegration form in 1 mL DI water** |
| --- | --- | --- | --- | --- | --- | --- | --- | --- | --- | --- |
| 1 | 2.2% |  | 8.9% | 44.4% |  |  | soft | residue*^b^* | <10 | liquid |
| 2 | 2.1% |  | 8.5% | 42.6% | 4.3% |  | chipped edges | residue | <10 | liquid |
| 3 | 2.0% | 11.8% | 7.8% | 39.2% |  |  | rough surface | clean | <10 | liquid |
| 4 | 1.6% | 9.5% | 6.3% | 47.6% | 3.2% |  | chipped edges | residue | <10 | liquid |
| 5 | 2.1% |  | 8.5% | 42.6% |  | 4.3% | hard | clean | ~60 | gel |
| 6 | 2.0% |  | 7.8% | 39.2% | 11.8% |  | chipped edges | residue | <10 | liquid |
| 7 | 1.5% |  | 6.2% | 61.5% |  |  | soft | residue | <10 | liquid |
| 8 | 1.2% |  | 4.7% | 70.6% |  |  | Soft | residue | <10 | liquid |
| 9 | 2.2% |  | 8.7% | 43.5% |  | 2.2% | hard | clean | <10 | gel |
| **10*^c^*** | **2.2%** |  | **8.8%** | **44.0%** |  | **1.1%** | **hard** | **clean** | **<10** | **liquid** |

^a^ Abbreviations: MCC: microcrystalline cellulose; NFDM: nonfat dry milk; β-CD: 2-hydroxypropyl-beta-cyclodextrin; CMC: carboxymethyl cellulose;

DT: disintegration time; s: seconds; DI: deionized water.

*^b^* Residue in blisters post removal of tablet indicates loss in product.

*^c^* Formulation 10 was the successful formulation reported in this paper.
